# Placental Volume and Uterine Artery Doppler in Pregnancy Following In Vitro Fertilization: A Comprehensive Literature Review

**DOI:** 10.3390/jcm11195793

**Published:** 2022-09-29

**Authors:** Serena Resta, Gaia Scandella, Ilenia Mappa, Maria Elena Pietrolucci, Pavjola Maqina, Giuseppe Rizzo

**Affiliations:** Department of Obstetrics and Gynecology, Fondazione Policlinico Tor Vergata, Università di Roma Tor Vergata, Viale Oxford 81, 00133 Roma, Italy

**Keywords:** in vitro fertilization, placenta uterine Doppler, fetal growth restriction pre-eclampsia

## Abstract

The number of pregnancies achieved using in vitro fertilization (IVF) is rapidly increasing around the world. The chance of obtaining a successful pregnancy is also significantly improved due to technological advances and improvement in infertility treatment. Despite this success, there is evidence that pregnancy conceived by IVF has an increased risk of adverse maternal and perinatal outcome mainly represented by the development of hypertensive diseases, pre-eclampsia, and fetal growth restriction. Although different cofactors may play a role in the genesis of these diseases, the development of the placenta has a pivotal function in determining pregnancy outcomes. Advances in ultrasound technology already allows for evaluation in the first trimester, the impedance to flow in the uterine artery, and the placental volume using Doppler and three-dimensional techniques. This review article aims to describe the modification occurring in placental volume and hemodynamics after IVF and to summarize the differences present according to the type of IVF (fresh vs. frozen-thawed embryos).

## 1. Introduction

In the past few years, the percentage of pregnancies obtained from in vitro fertilization (IVF) is dramatically increasing, overall due to an implementation of new technologies and to the relevant percentage of infertile couples in reproductive-age estimated to be between 8 and 12% worldwide and of 15% in Italy [1,2]. An increase in the number of couples that resorted to ART has been registered, going from 77.509 in 2018 to 78.618 in 2019 [2].

An increase in adverse obstetrical outcomes after IVF compared to natural conception has been widely studied, above all placenta-related pregnancy complications such as: placental insertion abnormalities (placenta previa, placental abruptio, placenta accrete) and short-term and long-term placenta-related diseases. The former includes preeclampsia (PE) abnormality in fetal growth causing a small for gestational age (SGA) fetus and fetal growth restriction (FGR) or accelerated resulting in a large for gestational age (LGA) fetus. Short term placental related disease includes preterm birth (PTB) and postpartum hemorrhage [3,4,5]. Long term placenta-related disease manifests in adulthood and includes cardiovascular disease, metabolic syndrome, diabetes, and obesity [6,7]

The impact of different procedures as elective frozen-thawed embryo transfer (eFET) and fresh embryo transfer (ET) on the pregnancy rate and outcomes was extensively studied and there is evidence that pregnancies from frozen embryos had lower obstetric and perinatal complications when compared those obtained after fresh oocyte cycles in terms of a decreased rate of SGA, and ovarian hyperstimulation (OHSS). No difference in the rate of live birth between the two strategies (eFET and fresh ET) was found, while a higher prevalence of LGA fetuses and maternal hypertension in hormonal treatment cycle eFET was described [8,9,10]. Thus, the transfer of eFET is nowadays considered a standard procedure in many fertility clinics [8].

Despite these findings, the underlying mechanisms causing higher risk in adverse obstetrical outcomes in pregnancies obtained from IVF are not yet fully clarified. Poor pregnancy outcome has been related to a defective early placentation occurring at different levels, either in the restricted remodeling or in obstructive lesions of the spiral arteries. There are different factors that might cause an impaired trophoblastic invasion and influence placental development such as the impaired endometrium receptivity linked to hormonal therapy, the epigenetic modifications in the embryo related to IVF procedures, maternal immune response, or different cryopreservation procedures [11,12,13].

In this way, studying the development of placenta in the IVF-pregnancies is becoming a priority in the research agenda. A correct development of the placenta is a prerequisite for the pregnancy progress and studying placental development during pregnancy has become challenging.

The ultrasound has allowed for investigation of the development of the placenta through some variables, such as the evaluation of the placental volume by using three-dimensional (3D) ultrasonography and the evaluation of the impedance to flow in the uterine arteries (UtA) by calculating the pulsatility index (PI) with Doppler. In IVF-pregnancies, the evaluation of these variables promises to be a useful tool for early detection of placenta-related disorders [14,15].

The aim of this review is to provide to readers an update on the impact of IVF on obstetrical and perinatal outcomes in the attempt to clarify if the first trimester ultrasonographic variables may be applied in the prediction of PE and anomalies of fetal growth in such pregnancies. The identification of high-risk pregnancies is of paramount importance as these women could benefit from tighter follow-up and dedicated management to avoid or to reduce maternal and fetal morbidity conditions. 

## 2. Obstetric and Perinatal Outcomes Resulting from Ivf Pregnancies

The placenta must guarantee the maintenance of the pregnancy and the fetal well-being through correct exchange of gases, growth factors, endocrine signals, cytokines, and nutrients. Placenta development starts at approximately 6–10 days post-conception, when trophoblast cells of blastocyst adhere to the decidua [16]. In early gestation, the human placenta is constituted by two layers: an inner one of proliferating cytotrophoblasts, that ensures the exchange of nutrients and oxygen from maternal blood and an outer which assures a correct amount of blood during the pregnancy by invading the endometrial stroma and remodelling the uterine spiral arteries [17]. The placentation process represents a complex and not fully understood process of immunotolerance: during the adhesion and invasion of the myometrium by the blastocyst, an immunomodulation release of pro-angiogenic and endothelial factors happens, which leads to adaptive changes of the uterine spiral arteries [18]. New studies are focusing their attention on the origins of placental mesenchymal cells as they appear to have a pivotal role in establishing and sustaining the development of placental vasculature [16,17,18,19]. Despite its importance in the success of reproduction, the development of the human placenta is yet to be fully understood despite an altered placentation could lead to miscarriage, unexplained stillbirth, preterm labor, placental abruption, PE, and fetal growth anomalies [17,18,19,20,21,22].

Despite the improvement occurring in laboratory technology and clinical management of infertile women requiring IVF, this procedure is still associated with an high rate of adverse perinatal outcomes and overall placenta-related pregnancy complications [23,24,25].

Recent meta-analysis studies have confirmed how pregnancies obtained from IVF techniques are associated with an increased risk of poor obstetric outcome including: miscarriage, chromosomal abnormalities, PE, PTB, FGR), placenta previa, abruptio placentae, post-partum haemorrhages, as well as peri and postnatal complications, such as neonatal death, low birth weight infants, congenital malformations, musculoskeletal abnormalities and childhood cancers [26,27].

The risk of obstetric complications can be largely increased by many factors: presence of twin pregnancies after multiple embryo transfers, as well as an older pregnant population and gametes quality, previous history of recurrent abortions (RPL) and causes of infertility itself (polycystic ovarian syndrome) [10,28]. Unfortunately, only little data are available on the explanations of such augmented risk. Different mechanisms have been assumed to play a role in the defective early placentation including genetic and epigenetic mechanisms of implantation, alterations in endometrial receptivity, invasion, and growth of the trophoblast, genetic and/or epigenetic alterations of oocyte and/or embryos due to biological manipulations, and immunotolerance in case of egg donor pregnancies [29]. 

Recent meta-analyses have proved that in singleton IVF pregnancies there is an increased risk of placental abruption (RR 1.83, 95% CI 1.49 to 2.24), placenta previa (RR 3.71, 95% CI 2.67 to 5.16), antepartum (RR 2.11, 95% CI 1.86 to 2.38), and postpartum hemorrhage (RR 1.29, 95% CI 1.06 to 1.57) [27,30]. A higher incidence of gestational hypertension and diabetes, cesarean deliveries, PTB, SGA, and perinatal mortality was also described [30]. Nevertheless, relevant biases were present due to the inclusion in the natural conception group of women who obtained the pregnancy with ovulation induction or intrauterine insemination, leading to an underestimation of the association between ART and adverse outcomes [30]. Consequently, the risk of developing gestational diabetes, placental abruption, PTB, fetal growth defect, and perinatal mortality may be further increased when the control group is constructed excluding these women from the spontaneous conception definition [30] and limit the recalculation of the odds ratio or relative risk of the maternal and perinatal complications occurring in IVF women.

Of interest is the lack of IVF specific pathologies and they resemble the same characteristics of when these diseases are present in a naturally conceived population. In other words, there are no different phenotypes of PE, FGR or placenta accrete spectrum between the 2 groups of women despite the higher prevalence in the IVF group.

### 2.1. The Role of Ovarian Stimulation

Concerning safety-evaluation in IVF it is necessary to highlight the difficulties in discerning the influence on the outcomes that the underlying causes of infertility might bring versus potential risks related to IVF procedures themselves. IVF are characterized by the ovarian hormonal stimulation followed by the pick-up of the oocytes and their subsequent fertilization. This procedure implies the transfer of a single or fresher or eFET embryos. eFet embryos after thawing may be transferred in the uterus during natural or hormonally artificial induced cycles. In recent years, the number of eFET has increased and so have pregnancy rates, which are now better than those following fresh IVF embryos transfer [31].

It has been suggested that controlled ovarian stimulation (COS) (e.g., subcutaneous gonadotropins) lead inevitably to a change in the maternal hormonal structure, determining changes in the woman’s reproductive system, as modifications of the endometrium. Different hormonal treatment strategies used in controlled ovarian stimulation and laboratory IVF techniques can negatively impact endometrial receptivity and gamete status. Hence, it was suggested that performing eFET was better than fresh embryo transfer being associated with decreased ovarian hyperstimulation incidence with improved reproductive outcomes [32]. However, it is still unclear how the different preparation methods of the endometrium can affect the outcomes of eFET pregnancies and the selection of the treatment of choice [33].

### 2.2. Differences between Fresh and Freeze and Thawed Embryo Transfer

Two recent systematic reviews and meta-analyses demonstrated that singleton pregnancies obtained from eFET show a more favorable maternal and perinatal outcomes than those reached after fresh oocyte transfer including a lower risk of PTB (<37 weeks) (RR 0.84, 95% CI 0.78 to 0.90), SGA (RR 0.45, 95% CI 0.30 to 0.66) and birthweight <2500 g) (RR 0.69, 95%CI 0.62 to 0.76). The incidence of perinatal mortality, antepartum hemorrhage, congenital anomalies, and admission to neonatal units resulted similarly between the two procedures. Conversely, a large, randomized trial demonstrated a higher risk of delivering LGA newborns and the development of hypertensive disorders of pregnancy in the eFET group [31]. Probably, these discoveries suggested that the hyperestrogenism following controlled ovarian stimulation in fresh ET, immediately before embryo implantation, might lead to abnormal endometrial angiogenesis resulting in a reduced implantation and altered placentation. Conversely, hormonal levels in eFET cycle could recreate a more natural uterine environment [34]. However, the underlying mechanisms suggesting a greater incidence of LGA babies in eFET are still to be clarified. Possible explanations should be a better implantation potential, better placentation, and subsequent fetal overgrowth or epigenetic modifications in the early embryonic stages due to freezing and thawing procedures [35].

Unfortunately, there is a heterogeneity among studies, which made their comparability difficult in terms of population sampled, design of the studies, freezing methods (slow freezing or vitrification), embryo stage, natural cycles, or hormone replacement used [9,36]. Furthermore, the results of these meta-analyses were based on observational studies, making them subject to bias. 

In contrast with previous Roque M et al. [24] in a recent meta-analyses analyzing 11 randomized controlled studies including 5379 patients showed no difference in rates preterm birth between fresh ET and eFE a result different from that previously reported [9,36]. It also showed a significant increase in live birth rates (LBTs) with eFET solely in hyper-responders patients and in pregnancies undergoing PGT-A. Further, this study confirmed the risk of pre-eclampsia was higher with eFET compared to fresh ET, probably due to endometrial priming with supraphysiological concentrations of estrogen during artificial FET cycles. This conclusion is in agreement with the result of a recent Cochrane review, showing a lower prevalence of ovarian hyperstimulation syndrome in eFET cycle despite no difference in the cumulative life birth ratio between the two strategies [8]. This explains why the transfer of frozen-thawed embryos has become the standard procedure in most fertility clinics. Although this procedure does not seem to reduce IVF success rates, an increased prevalence of PE after eFET technique has been reported [8,37]. 

### 2.3. Endometrial Preparation

Endometrial preparation for an embryo transfer (e.g., oral estradiol and luteal phase support) may influence the endocrine uterine environment during the embryo transfer, playing an essential role in vascular adaptation of the mother to pregnancy, increasing the risk of placental development and weight of the offspring. 

Endometrial preparation before eFET can occur how ovulatory or programmed cycles. To date, in frozen embryo transfer there is no consensus on the best endometrial preparation method or the duration of hormonal replacement [38]. Emerging data suggests that these differences could have a detrimental impact on adverse obstetrical outcomes in pregnancies from artificial cycles, above all in hypertensive disorders [33]. 

In 2019 Saito et al. [6] evaluated the pregnancy outcomes of 100,000 patients undergoing FET during natural or hormonal replacement cycles. Pregnancies conceived in a hormone replacement cycle had higher odds of hypertensive disorders of pregnancy (4% vs. 3%, aOR 1.43; 95% CI, 1.14–1.80), placenta accreta (0.9% vs. 0.1%, aOR 6.91; 95% CI, 2.87–16.66) cesarean section (44.5% vs. 33.7%, aOR 1.69; 95% CI, 1.55–1.84) and post term delivery associated with a decreased risk to develop gestational diabetes mellitus (1.5% vs. 3.3%, aOR 0.52; 95% CI, 0.40–0.68) in comparison to natural cycle FET. 

In agreement with these results, Ginström Ernstad et al. [39] in a large retrospective study, found an increased risk of hypertensive disorders in pregnancy (10.5 vs. 6.1%, aOR 1.78; 95% CI 1.43–2.21) and postpartum hemorrhage (19.4% vs. 7.9%, aOR 2.63; 95% CI, 2.20–3.13) in hormone replacement cycles when compared to natural cycles. Moreover, higher risks for post-term birth, macrosomia, and cesarean delivery were detected [39].

Given that endometrial preparation is a less physiological condition than a natural cycle, the increased risk of hypertensive disorders may be due to changes in endometrial receptivity modulating placental development. Moreover, it was hypothesized that in patients who have programmed cycles have a decrease of substances produced by the corpus luteum in early pregnancy, particularly the potent vasodilator relaxin and vascular endothelial growth factor levels, lower angiogenic and nonangiogenic circulatory endothelial progenitor cells and a lack of drop in mean arterial pressure during pregnancy [40,41]. It was demonstrated that the CL is implicated in the adaptation of the maternal cardiovascular system in early gestation and its absence in eFET may be associated with reduced aortic compliance and increased risk of PE [40,41]. Anyway, the association between endometrium preparation and adverse obstetric outcomes must be clarified with further studies that include other possible confounders.

Indeed, every single step or procedure carried out during IVF can play an independent and essential role in determining obstetric risks: cryopreservation methods, different hormonal treatment, and laboratory techniques. Vitrification showed higher pregnancy rates than slow-freezing, however perinatal outcomes are similar between the two methods [42,43]. Potential impact of gamete manipulation, as intracytoplasmic sperm injection (ICSI) and in vitro embryo culture were investigated in recent literature. Specific laboratory procedures, such as incubation systems, types of embryos culture used, the duration of the culture, and ICSI could constitute a source of “stress” for the developing embryo. At last, further large studies are required to identify the contribution of each single confounder on pregnancy and obstetrical outcomes after ART.

## 3. Non-Invasive Parameters in the First Trimester of Placental Development In-Vitro Fertilization Pregnancies

As previously mentioned, the inadequate trophoblastic invasion seems to be the most important etiological factor in the early-onset PE and FGR [44]. Given the increase in the number of pregnancies achieved with IVF, the prediction and possible prevention of adverse outcomes in such women is clinically relevant. 

### 3.1. First Trimester Uterine Doppler

The assessment of placental development during pregnancy is challenging but can be assessed by evaluating some first-trimester non-invasive parameters such as the impedance to flow in the uterine arteries by calculating the UtA-PI and the assessment of first trimester placental volume (PV) and utero-placenta vascular volume (uPVV). 

**Figure 1:**
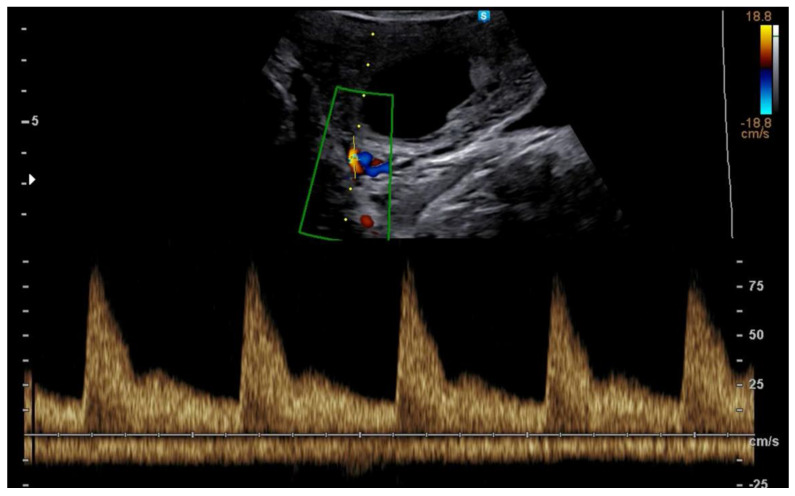
Example of Doppler tracing obtained at 12 weeks from the uterine artery.

In a spontaneously conceived pregnancy, there is a decline of placental vascular resistance resulting in a progressive decrease of UtA-PI in the three trimesters of pregnancy (Figure 1) [45]. 

An impaired trophoblastic invasion of the uterine decidua indices an altered remodeling of the spiral arteries determines an increased vascular resistance in the uterine arteries already evident from 11 weeks onwards and it is frequently associated with a later development of PE [46,47,48]. Therefore, given the potential consequences of a higher incidence of placenta-related adverse outcomes in IVF pregnancies, the evaluation of impedance to flow in the uterine arteries in the context of in vitro fertilization was of particular interest. 

Despite the high incidence of PE in IVF women, no difference was found in UtA-PI when compared with natural conceived pregnancies in the first trimester. On this basis there are extensive reports suggesting that the underlying mechanisms behind the increased incidence of PE is not related to an impaired uteroplacental perfusion [49,50,51]. It might be due to a coexistence of different factors that lead to abnormal placental development, such as different expression in placental gene expression or the presence of an abnormal immune response at the maternal–fetal interface that takes place particularly when the pregnancies are obtained with egg donor [13,52,53].

Few studies have evaluated UtA-PI in IVF patients comparing between pregnancies conceived from eFET and fresh blastocyst transfer [49,50,51]. Two studies showed a better uterine perfusion and fetal growth in the frozen blastocyst transfer group compared to those that underwent fresh blastocyst transfer [3,54].

Choux et al. showed that PI was significantly higher in the fresh embryo transfer group (1.86 ± 0.64) than in the naturally conceived (1.52 ± 0.59; *p* = 0.001) and Pi was lower in the eFET group compared to the fresh embryo transfer group (*p* = 0.001) [3]. These results were confirmed by two other studies that observed lower UtA-PI values for the eFET group compared with the fresh-blastocyst-transfer group [54,55].

Differences in maternal characteristics and the IVF procedures used could explain the apparent contradiction of reduced UtA-PI in the eFET group during pregnancy, known to have a higher incidence of early-onset PE [56]. Instead, the higher risk of LGA and the lower risk of SGA could be explained from a lower UtA-PI in eFET. 

### 3.2. First Trimester 3D Placental Volume

**Figure 2:**
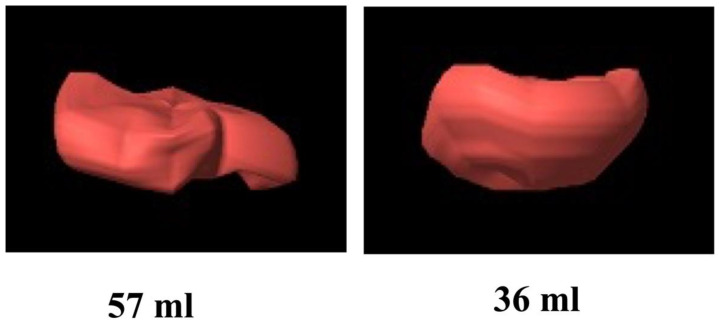
Example of 3D reconstruction of the placental volume at 12 weeks in a spontaneously conceived pregnancy and in an IVF with fresh embryo. The volume is significantly reduced in the latter.

A huge advance was made with the introduction of three-dimensional ultrasound, making it easier to measure placental volume. The implementation of three-dimensional (3D) ultrasound allowed for reproducible measurements of placental volume and has been shown to be an indicator of placental insufficiency, predicting the placenta-related pregnancy complications, such as PE (Figure 2) [48,57,58,59].

In IVF pregnancies, placental volume in ultrasound has been investigated and the results compared with that of the naturally conceived were conflicting [50,51,60,61,62,63]. Rifouna et al. [60] analyzed 70 pregnancies and no difference in placental vascular and trophoblastic volume in the first trimester was found between IVF and spontaneous pregnancies Rizzo et al. [51,63] reported significantly reduced placental volume in IVF pregnancies compared to spontaneous pregnancies, particularly in donor oocyte recipients, probably due to different immune responses of the mothers to trophoblast antigens. 

These discrepancies may be due to different techniques in performing ultrasounds, the largest samples in Rizzo’s study (70 versus 416) and differences among studies in the characteristics of IVF pregnancies [60,61,62,63].

To the best of our knowledge, only two studies analyzed placental volume and uterine artery Doppler distinguished IVF after fresh embryo transfer from those after eFET [3,51]. Rizzo et al. [51] found no differences in UtA-PI between frozen-thawed ET, fresh ET, and natural conception in agreement with other authors [49,50]. Furthermore, this study demonstrated the presence of a reduced placental volume in IVF pregnancies compared to those conceived naturally and the IVF pregnancies with fresh embryos showed a significantly lower placental volume than in the frozen-thawed embryos and a higher incidence of PE. It was hypothesized that altered endometrial receptivity due to the use of high-dose gonadotrophin ovarian stimulation in the fresh group could influence the placental development. As with Rizzo’s study, Choux et al. [3] found a larger placental volume in pregnancies after eFET compared to pregnancies after fresh embryo transfer. As placental volume correlated to birthweight, this is consistent with the findings of a higher incidence of LGA newborns after frozen-thawed ET [9,35].

A summary of the characteristics and the results obtained in the studies considered is reported in the Supplementary Material.

A possible explanation, as Conrad’s theory suggests [40], could be that the role of the corpus luteal is pivotal for a natural maternal hormonal environment during implantation and hemodynamic adaption to pregnancy and in this study, approximately 75% of frozen-thawed ET were performed in a natural cycle, in the presence therefore of a corpus luteal.

Future studies are needed to assess the clinical utility of first trimester vascularization indices and placental volume as a predictor of pre-eclampsia in IVF pregnancies [64].

## 4. Conclusions

This review confirms that pregnancies obtained with IVF have a higher incidence of maternal and perinatal adverse outcome than naturally conceived pregnancies. Among IVF pregnancies those obtained by eFET showed better obstetric and perinatal outcomes than those obtained after fresh oocyte cycles in term of lower risk of SGA, LBW, and ovarian hyperstimulation. Despite the absence of difference in the cumulative live birth rates between the two conception modes, there is a higher risk of hypertension disorders in hormonal treatment cycle in frozen-thawed ET. 

In this review, we were unable to clarify the underling mechanisms causing the maternal and perinatal complications due to the heterogenicity of the available studies on this topic and the impossibility of obtaining direct analysis on human pregnancies. Irrespective of these limitations, the higher risk of PE in eFET contrasts with the discovered that the measurement of placental volume in 3D ultrasound was lower in fresh embryos compared to frozen-thawed embryos. Often the main limitations of these studies were related to a lack of comparability due to a high risk of selection bias, such as the women’s characteristics, endometrial preparation, method of cryopreservation, and study populations.

Moreover, the potential clinical benefit should be underlined. Acquisition during the first trimester of uterine Doppler and placental volume allows for the identification of a subgroup of IVF women at a higher risk of developing complications for which a closer surveillance is necessary and in which prophylactic treatment can be applied under prospective multicenter trails

## Data Availability

Not applicable.

## Supplementary Materials

The following supporting information can be downloaded at: https://www.mdpi.com/article/10.3390/jcm11195793/s1.
