# Supplementary material for: Placental Volume and Uterine Artery Doppler in Pregnancy Following In Vitro Fertilization: A Comprehensive Literature Review
Source: J Clin Med. 2022 Sep 29;11(19):5793. doi: 10.3390/jcm11195793 (PMC9573239; doi:10.3390/jcm11195793)
Supplement: Supplementary file 1 [file jcm-11-05793-s001.zip › jcm-1946638-supplementary.pdf]

Journal of
Clinical Medicine

MDPI

Review

Placental Volume and Uterine Artery Doppler in Pregnancy Following In Vitro Fertilization: A Comprehensive Literature Review

Serena Resta, Gaia Scandella, Ilenia Mappa, Maria Elena Pietrolucci, Pavjola Maqina and Giuseppe Rizzo *

Università di Roma Tor Vergata, Department of Obstetrics and Gynecology, Fondazione Policlinico Tor Vergata, Viale Oxford 81 00133 Roma, Italy
* Correspondence: giuseppe.rizzo@uniroma2.it

Citation: Resta, S.; Scandella, G.; Mappa, I.; Pietrolucci, M.E.; Maqina, P.; Rizzo, G. Placental Volume and Uterine Artery Doppler in Pregnancy Following In Vitro Fertilization: A Comprehensive Literature Review. *J. Clin. Med.* **2022**, *11*, 5793. https://doi.org/10.3390/jcm11195793

Academic Editors: Aldo E. Calogero and Claudio Manna

Received: 16 September 2022
Accepted: 27 September 2022
Published: 29 September 2022

Publisher's Note: MDPI stays neutral with regard to jurisdictional claims in published maps and institutional affiliations.

Copyright: © 2022 by the authors. Licensee MDPI, Basel, Switzerland. This article is an open access article distributed under the terms and conditions of the Creative Commons Attribution (CC BY) license (https://creativecommons.org/licenses/by/4.0/).

Supplementary Material: Characteristics of the studied considered

					UtA-PI			PV			uPVV		
					IVF			IVF			IVF		
Study	Country	Study Design	N of patients	ART technique distinction	Fresh embryo	FET	Control	Fresh embryo	FET	Control	Fresh embryo	FET	Control
Rifouna et al. 2014	Netherlands	Retrospective cohort sudy	154 (70 IVF)	Y Fresh/FET embryos	N/A			42.7, SD 15.9		41.2, SD 13.9	27.6, SD 16.9		24.8, SD 19.9
Rizzo et al. 2016	Italy	Prospective study	914(416 IVF)	Y autologus/ donor	Autologus: 1.1 (IQR 0.7-1.5) Donor: 1 (IQR 0.8-1.4)$^{+}$		1.00 (IQR0.7-1.4) $^{+}$	Autologous: 0.80 (IQR, 0.50–1.10) donor: 0.63 (IQR, 0.40 – 0.80) $^{+}$		1.04 (IQR), 0.81–1.22)$^{+}$	N/A		
Sundheimer et al. 2018	USA	Retrospective cohort sudy	3613 (427 IVF)	N IVF/ non-IVF techniques	N/A			IVF+nIVF: 56.6 ± 32.9		55.2 ± 32.6	N/A		
Choux et al. 2019	France	Retrospective single-centre case-control study	252(84 IVF)	Y Fresh/FET embryos	1.86 ± 0.64 °	1.26 ± 0.44°	1.52± 0.59*	4.40 ± 0.283	4.38 ± 0.37	4.52 ± 0.39	N/A		
Cavoretto et al. 2020	Italy	Prospective longitudinal study	367 IVF	Y Fresh/FET embryos	1.00 ± 0.29 *$^{+}$	0.86 ± 0.28 *$^{+}$	N/A	N/A			N/A		

Van Dujin et al. 2021	Netherlands	Prospective cohort study	214 (88 IVF)	Y Fresh/FET embryos	β -0.148 (-0.304, 0.009)	β -0.112 (-0.305, 0.080)	Reference	β 0.003 (-0.127, 0.134)	β0.002 (-0.160, 0.163)	Reference	β-0.052 (-0.207, 0.103)	β0.071 (-0.121, 0.263)	Reference

°p <0.05, *p <0.001 , FET: frozen-thawed embryo transfer, + MoM.
